# In a Class of Their Own – RXFP1 and RXFP2 are Unique Members of the LGR Family

**DOI:** 10.3389/fendo.2015.00137

**Published:** 2015-09-07

**Authors:** Emma J. Petrie, Samantha Lagaida, Ashish Sethi, Ross A. D. Bathgate, Paul R. Gooley

**Affiliations:** ^1^Department of Biochemistry and Molecular Biology, University of Melbourne, Parkville, VIC, Australia; ^2^Bio21 Molecular Science and Biotechnology Institute, University of Melbourne, Parkville, VIC, Australia; ^3^Florey Institute of Neuroscience and Mental Health, University of Melbourne, Parkville, VIC, Australia

**Keywords:** leucine-rich repeat-containing G protein-coupled receptors, LGR, RXFP1, RXFP2, GPCR

## Abstract

The leucine-rich repeat-containing G protein-coupled receptors (LGRs) family consists of three groups: types A, B, and C and all contain a large extracellular domain (ECD) made up of the structural motif – the leucine-rich repeat (LRR). In the LGRs, the ECD binds the hormone or ligand, usually through the LRRs, that ultimately results in activation and signaling. Structures are available for the ECD of type A and B LGRs, but not the type C LGRs. This review discusses the structural features of LRR proteins, and describes the known structures of the type A and B LGRs and predictions that can be made for the type C LGRs. The mechanism of activation of the LGRs is discussed with a focus on the role of the low-density lipoprotein class A (LDLa) module, a unique feature of the type C LGRs. While the LDLa module is essential for activation of the type C LGRs, the molecular mechanism for this process is unknown. Experimental data for the potential interactions of the type C LGR ligands with the LRR domain, the transmembrane domain, and the LDLa module are summarized.

## Introduction

The receptors for the peptide hormones H2 relaxin and insulin-like peptide-3 (INSL3) are unique members of the leucine-rich repeat-containing G protein-coupled receptors (LGRs) family (1). LGRs are class A G protein-coupled receptors (GPCRs) and are divided into three groups: types A, B, and C. Type A LGRs are receptors for the glycoprotein hormones follicle-stimulating hormone (FSH), luteinizing hormone (LH), and thyroid-stimulating hormone (TSH). Although the Type B LGRs were identified in 1998 (1, 2), LGR4-6 were only recently deorphanized as the R-spondin (Rspo) receptors (3, 4). These receptors have roles in stem cell differentiation and are associated with cancers affecting the gut. The identification of LGR7 in 2000 resulted in the formation of the third group, Type C (1). Soon after, LGR7 was joined by the receptor encoded by the *GREAT* gene (5), (LGR8), when the phenotype of the knockout mouse correlated with abnormal testicular descent noted in INSL3 knockout mice (6, 7). In 2002, LGR7 and LGR8 were deorphanized as H2 relaxin receptors (8). At this exciting time of GPCR deorphanization, the grouping of LGR7 [later defined as RXFP1 in Ref. (9)] and LGR8 (RXFP2) with the glycoprotein hormone receptors into the LGR family appeared to correlate with the known reproductive roles and tissue-specific expression of H2 relaxin and INSL3. H2 relaxin is a major circulating hormone produced by the corpus luteum and placenta with important roles in maintaining pregnancy and facilitating parturition [reviewed in Ref. (10)]. INSL3 is produced in testicular Leydig cells in males and follicular theca cells in the female ovary (11), and therefore, has central roles in fertility.

Almost 15 years since the initial identification of RXFP1, the landscape of H2 relaxin research is diverse and complex. H2 relaxin is considered a pleiotropic hormone with many functions, including central roles in collagen turnover (12, 13) wound healing (14), and roles in cardiovascular function (15) [further reviewed in Ref. (10)]. The key roles of relaxin in cardiovascular function lead to the use of the human form of relaxin, H2 relaxin, in clinical trials for the treatment of acute heart failure. With the success of these clinical trials (16–18), a clear understanding of the mechanism of how H2 relaxin binds and activates RXFP1 is highly desirable. Unfortunately, no structures of a Type C LGR are available. In this context, as structural understanding of other members of the LGR family grows, we review the structural knowledge of the LGR family, and examine what is known about ligand interactions at the extracellular domains (ECDs) of the Class C LGRs in comparison to the other members of this diverse family of GPCRs.

## Leucine-Rich Repeat-Containing G-Protein Receptor

The LGR family is classified as “Type A” rhodopsin-like GPCRs based on the similarity of its transmembrane (TM) domain. They are unified into this family based on their large ECDs containing leucine-rich repeats (LRRs). The first LRR-containing protein to be identified was leucine-rich α2-glycoprotein (LRG) (19) but since then, LRR domains have been identified in various proteins including extracellular, intracellular, and TM proteins with a wide variety of functions, such as neural circuit formation [reviewed in Ref. (20)], inflammation [reviewed in Ref. (21)], immune response against pathogen (22, 23), and development and immunity in plants (24).

### LGRs classification

The LGR family is differentiated on the basis of the number of LRRs within the ECD, the length of the hinge region between the LRR domain and the TM domain and the presence of a low-density lipoprotein class A (LDLa) module (25, 26). Currently, there are three types of LGRs: type A, type B, and type C (Table 1). Mammalian type A LGRs include the follicle-stimulating hormone receptor (FSHr), thyroid-stimulating hormone receptor (TSHr), and luteinizing hormone receptor (LHr) [or lutropin/choriogonadotropic receptor (LCGr)] (27). Type A are characterized by 7–9 LRRs within the LRR domain and have a distinctively long hinge region, connecting the LRR to the TM domain, which is essential for receptor activation (26). The type B LGRs (LGR4–6) are the receptors for the Rspo family (R-spondin1–4) and have roles in development, including cell proliferation and differentiation, and oncogenesis (28). These LGRs, typically have 16–18 LRRs and so constitute a longer LRR domain than type A and type C LGRs (26). The hinge region of type B LGRs is “medium length” compared to that of type A. Type C members are distinct in that they have an N-terminal LDLa module, which is also known to be important for receptor activation (29). These latter receptors include the mammalian LGR7 and LGR8 (now known as RXFP1 and RXFP2, respectively) along with a snail LGR and LGR3 and LGR4 from *Drosophila* (30) and are grouped as C1 or C2 based on the number of LDLa modules in their ECD. Type C LGRs have a similar number of LRRs compared to type A LGRs, although they have a shorter hinge region connecting the LRR domain to the TM domain (26). There is no evidence to suggest that the hinge has the same role in modulating receptor activity as it does in type A LGRs. RXFP1 and RXFP2 are the only mammalian class C LGRs and contain a single LDLa module, while type C LGRs found in echinoderm and molluskan can contain up to 12 modules (26). Thus, the evolution of these receptors is difficult to determine and in the context of this review only the mammalian RXFP1 and RXFP2 receptors will be discussed.

**Table 1:** **Ectodomains and ligands of the LGR family**.

Name	Short annotated name	Ligand	No of LRRs	Residues per repeat	Ligand affinity^a^	PDB
**Type A**
LGR1	FSHr	Follicle-stimulating hormone	9	21–25	0.03–3 nM^b^	1XWD, 4AY9, 4MQW
LGR2	LH/CGr	Lutropin or choriogonadotropic hormone	6	22–31	0.3–0.5 nM^c^	
LGR3	TSHr	Thyrotropin (thyroid-stimulating hormone)	7	20–31	0.25 nM^d^	3XWT,3GO4
**Type B**
LGR4	LGR4	Rspondin1–4	17	20–25	56 nM^e^	4KT1, 4QXE, 4QXF
LGR5	LGR5	Rspondin1–4	17	21–26	3 nM^f^	4BSR, 4BSS, 4BST
						4BSU, 4KNG
LGR6	LGR6	Rspondin1–4	17	21–25	0.5–7 nM^g^	
**Type C**
LGR7	RXFP1	H2 relaxin	10	24–25	9.2–9.8^h^	2JM4 (LDLa module)
LGR8	RXFP2	INSL3, H2 relaxin	10	24	9.3–9.7, 8.5–9.0^h^	2M96 (LDLa module)

*^a^Reported *K*_d_, unless noted*.

*^b^Simoni et al. (31)*.

*^c^Ascoli et al. (32)*.

*^d^Harfst et al. (33)*.

*^e^Wang et al. (34)*.

*^f^de Lau et al. (3)*.

*^g^IC_50_ (35)*.

*^h^pK_d_ or pK_i_ (36)*.

### Structural features of LRR domains

#### The LRR Domain

The first structure of a LRR-containing protein, ribonuclease inhibitor (RI), showed a horseshoe-shaped structure (37). This curved structure consists of a β-sheet on the concave side of the LRR and an array of α-helices on the convex side. A single LRR consists of a β-strand and α-helix connected with loops and therefore a sequence of LRRs forms alternating parallel β-strands and α-helices along the α/β fold (38). The β-strand is formed by a highly conserved motif, xLx, within a LRR, connected to adjacent parallel β-strands by hydrogen bonds to form the β-sheet on the concave side of the structure. Comparison of LRR domains show the presence of a repeated conserved hydrophobic-rich sequence motif, LxxLxLxxNxL, where the underlined residues form the β-strand, x is any amino acid and leucine may be substituted by valine, isoleucine or phenylalanine; and asparagine by cysteine, serine or threonine (38, 39).

The convex side of the LRR domain is comprised of more variable sequence and secondary structure including 3^10^ helices, polyproline II helices, β-turn or β-strand (39). In addition to the length, the nature of the sequence contributes to the curvature of the LRR domain. Two distinct sequences are observed on the convex side, LPxxL (LP motif) and IxxxAF (AF motif) (40). The prototype LRR protein comprising the LP motif is the platelet-receptor glycoprotein Ibα that has a steep curvature (41), whereas the prototype AF motif is the Nogo receptor which has a relatively flat curvature (42). The LRR domain is an exceptionally stable solenoid-like structure. The side chains of the leucine residues (or other aliphatic residues) are closely packed and oriented toward the interior of the domain to form a hydrophobic core in a similar fashion as observed in other globular proteins (39). The β-sheet along its concave side also contributes to the stability of the structure as each β-strand forms five hydrogen bonds to the adjacent β-strand. To further stabilize the structure, the conserved asparagine residues (on the concave side) form an asparagine ladder where the side chains stack on top of adjacent asparagine residues and form hydrogen bonds (39, 43). For LRR proteins with repeating AF motifs, the phenylalanines on the convex side form a phenylalanine spine that also adds to the stability of the LRR domain (42, 44). Commonly, a binding site is located in the concave surface of the LRR domain, however, the convex surface also can be utilized as site of ligand interaction (39).

#### The N- and C-Terminal Caps

Although the LRR domain is a stable solenoid structure, it would appear that capping structures are essential to maintain stability. In most cases, especially extracellular LRR and membrane-associated LRR proteins, there are cysteine-rich subdomains at the N- and C-terminal ends of the LRR domain, termed N-terminal (LRRNT) and C-terminal (LRRCT) capping motifs, respectively. Based on sequence analysis, LRRNT motifs have a consensus sequence of CPx(2-5)CxCx(6-19)Cx(6-8)Px(3)Px(5)LxL, where x indicates any residue (39, 45). The typical structure of LRRNT contains a β-strand antiparallel to the main LRR β-sheet, followed by 20 to 21 residues before entering a β-strand that is parallel to the LRR. As this strand is often not a canonical LRR, it is excluded from the description of the body of the LRR domain.

Based on phylogenetic analysis and the number of cysteine residues present, there are four types of LRRCT motifs, CF1–4 (45). CF1 is the most common capping structure containing four cysteines (CxCx(17–24)Cx(9–18)CxxP). CF2 has two cysteines, separated by 33 to 34 residues and is found in small proteoglycans, CF3 has three cysteines (CCx(14–27)C) found in GPCRs, and CF4 has two cysteines separated by 1 to 11 residues and is found in plant LRR proteins.

## Structures of LGRs

### Type A LGRs

**Figure 1:**
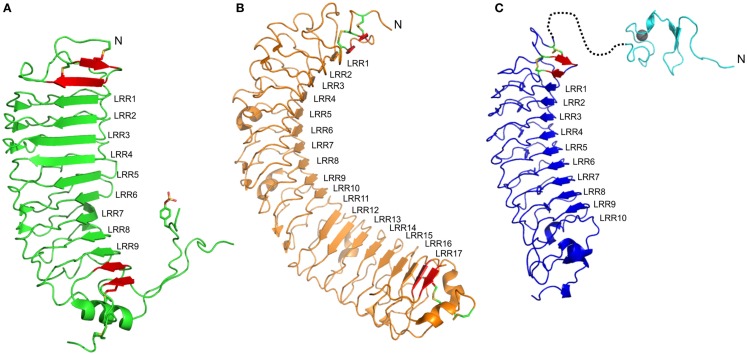
**Structures of ectodomains of members of the LGR family**. **(A)** The type A member FSHr (PBD: 4AY9) shows nine LRRs. LRR1–6 show a shallow curvature while the dominance of LP motifs in the convex side of LRR7–9 results in a steep curvature. The ligand shows interactions to most of the LRRs, especially LRR1–5 and LRR7–9 **(B)** The type B member LGR4 (PDB: 4KT1). The concave side of the LRR protein is separated into two sheets, LRR1–10 and LRR11–17, due to the absence of the conserved Asn residues within the LRR motif of LRR11 and 12. The ligand binds to the first sheet, making contacts with residues in LRR1, LRR3–9. **(C)** A homology model of the ECD of the type C member RXFP1. The 10 LRRs are predicted to form a shallow curvature. The ligand, H2 relaxin, is expected to bind to LRR4–6 and LRR8. The structure of the N-terminal LDLa module (PDB: 2JM4) for this ECD is also shown, although the structure of the linker that joins to the LRR domain remains unknown. In each structure, additional β-strands (red), which are integral to the domain, are shown but these strands typically lack the xLx portion of the LRR motif, and usually include disulfide bonds characteristic of the N- and C-terminal capping motifs. At the N-terminal end of each LRR domain, an antiparallel β-strand followed by a β-strand parallel to the remainder of the LRR is observed. At the C-terminal end, significant differences for the three members are observed. For FSHr, a large hinge containing a functionally important sulfated Tyr residue is present; for LGR4, this hinge is absent, but a typical CF3 capping motif is present; for RXFP1, the C-terminal cap does not appear conserved, the hinge is short, and therefore, the structure of this region is difficult to predict.

The FSHr crystal structure is the best understood of the LGRs (46). The LRR domain consists of repeats of irregular length and conformation (Figure 1A). As expected the LRR domain contains an LRRNT with an antiparallel β-strand followed by the expected parallel β-strand of this cap. This is then followed by nine parallel β-strands of the LRR domain (Table 1), and additional two parallel β-strands in the C-terminal cysteine cap, which form a typical CF3 cap. Prior to the last parallel β-strand, there is an insertion of an α-helix and a long hairpin loop that contains a sulfated tyrosine, collectively referred to as the hinge region, and forms an integrated structure within the LRR domain (Figure 1A) (47). Consequently, the entire LRR domain consists of 12 parallel β-strands. On the convex side of the LRR domain, there are seven short β-strands separated into three β-sheets. Importantly, the intervening sequences of the convex side follow from the N-terminal end as: an LP motif, three AF motifs, one LP motif, two AF motifs, and then three LP motifs. Thus, there is an increasing curvature of the domain running from N- to C-terminus. Superimposing the structures of the FSHr and TSHr LRR domains shows similar structures despite different primary sequences and disulfide connectivity (40).

Upon binding to ligand, there is a significant change to the hinge structure of FSHr, otherwise the LRR domain is similar to that of the ligand free (47). The binding of FSH is described as a “handclasp” interaction, where 10 parallel β-strands of FSHr, including the parallel β-strand of the LRRNT, are in contact with the hormone mainly via electrostatic interaction (46). The interactions can be divided into two flat surfaces; one is where the C-terminal end of the α- and β-units of FSH interact with the parallel β-strand of the LRRNT and the first six LRRs and the other is where the second loops of the α- and β-units of FSH interact with the tips of the β-strands of LRR1–5 and the C-terminal ends of LRR7–9, respectively. The hairpin loop between the last two parallel β-strands presents an essential tyrosine residue, which becomes sulfated (sTyr). This sTyr makes an important contribution to ligand binding by inserting into a hydrophobic pocket in FSH. As it is clear that FSH can bind in the absence of this hinge region, this structure provides evidence of a “two-step” binding mechanism (47). Following FSH binding to FSHr, the sTyr binds to the hormone lifting the hairpin loop from an inhibitory state of the TM domain that results in activation of the receptor.

The LRR domain and the ligand of FSHr and TSHr are similar, although the disulfide arrangement of the LRRNT differs (48). Nevertheless the mechanism is suggested to be the same for these receptors as TSHr has a structurally similar sTyr site (49, 50), which is essential for TSH binding and activation (51–53). Currently, there is limited knowledge about the activation mechanism of LHr. Although the sTyr site is present in the hinge region of LHr (54, 55), the mechanism of LHr differs from FSHr and TSHr as removal of the ectodomain does not result in constitutively active receptor (56).

### Type B LGRs

The LRR domains in Type B LGRs are typically larger than those of Type A LGRs (Table 1). The crystal structures of both LGR4 and LGR5 comprise 17 typical LRR β-strands with an N-terminal antiparallel β-strand followed by another β-strand that is parallel to the LRR domain, and two additional parallel β-strands at the C-terminus (Figure 1B) (34, 57). On the convex side, the secondary structures are more variable with various lengths of loops, α-helices, and β-strands. In LGR4 and LGR5, the conserved asparagine residues in LRR11 and LRR12 are missing, resulting in two separate β-sheets, one from LRR1–10 and the other from LRR13–17 (Figure 1B) (34, 58–60). The intervening sequences on the convex side follow from the N-terminal end: in the first β-sheet, two AF, four LP, one AF, two LP, and one AF; and in the second β-sheet, three LP, two AF, and one LP motif. The large number of LP motifs result in a more curved surface than the type A receptors. These proteins have a typical LRRNT, whereas the LRRCT has a short four-residue intervening sequence within the otherwise CF3 cap.

Recently, Rspos were identified as the native ligands of type B LGRs (4, 61, 62). In LGR4, the ligand binds to the first LRR and LRR3–LRR9 (the first β-sheet) and the binding interface is smaller compared to FSH–FSHr (1860 compared to 2600 Å^2^) (34, 60). The interface is mainly electrostatic within LRR4 and hydrophobic within LRR5–7. Similar to LGR4, the LGR5 binding interface consists of LRR3–9 with total surface area of 870 Å^2^ (58). LGR5 binds to Rspo in a similar manner to LGR4, a mix of charged and hydrophobic clamping interactions. Based on these observations, the binding of Rspo is conserved across the type B LGRs and it is supported by the fact that there is lack of specificity between different Rspos and type B LGRs (3). In type B LGRs, there is no evidence that the hinge region or even the LRRCT is involved in ligand binding or activation. The LRRCT of LGR4 can be deleted or substituted with LRRCT motifs from other proteins without affecting activity or binding (63). Moreover, antibodies targeted to the LRRCT of LGR5 do not block Rspo activity (62). However, another antibody targeted specifically to the hinge region has been shown to induce activity in the absence of Rspo (58).

While there are similarities in how type A and type B LGRs bind ligand, the signal pathways and receptor activation are different. Rspo signaling mediated by LGR4, LGR5, or LGR6 is through Wnt signaling and not the canonical GPCR pathways (3, 61, 63), which is in contrast to type A and type C LGRs. Multiple mechanisms have been proposed to explain how type B LGRs regulate Wnt signaling. The binding of Rspo to the ectodomains of LG4–6 recruits the E3 ligases (RNF4 and ZNRF3) to form a ternary complex that promotes clearance of the E3 ligase, thus a reduction in Wnt receptor ubiquitination and degradation, and consequently increased Wnt signaling (64). In addition, the Rspo–LGR4 recruits the scaffold protein IQ motif containing GTPase-activating protein 1 (IQGAP1) into the Wnt complex to potentiate signaling (65).

### Type C LGRs

Presently, there are no structures of the LRR domains of type C LGRs. However, the structures of type A and type B LGRs, as well as those of other LRR domains, allow predictions to be made for this class of LGRs. Based on the primary sequence, the LRR domain is expected to have 10 LRR repeats (Table 1) with an N-terminal antiparallel β-strand and an additional parallel β-strand forming the N-terminal cap. Analysis of the LRRs from the type C LGRs, RXFP1 and RXFP2, suggests that the LRRs are more regular than the type A and type B LGRs. The intervening sequences on the convex side of RXFP1 following from the N-terminal end are predicted to be four AF, one LP, two AF, and one LP motif. Such an arrangement predicts a relatively flat surface and the predominance of the AF motif allows straight forward homology modeling of the LRR domain of RXFP1 based on the prototype AF motif Nogo receptor (42). A homology model built by Modeler (66), using the Nogo receptor (PDB: 1OZN) as a template with ~29% sequence identity, shows a spine of phenylalanine residues down the convex side, except at LRR5 where a leucine residue is aligned, and a ladder of asparagine residues of the LRR motif on the concave side of the model (Figure 1C). The conservation of the N-terminal cysteine residues predicts an LRRNT similar to type A and type B LGRs. However, there are only two cysteine residues in the C-terminal hinge of RXFP1, separated by eight residues, and therefore are CF4-like rather than the CF3 capping motif expected in LRR-containing GPCRs (45). Therefore modeling of this region against the Nogo receptor, or any other LRR protein, is highly speculative. The hinge region of RXFP1 and RXFP2 is relatively short (~30 residues compared to 72–123 residues in other LGRs) (1). Considering the shortness of the hinge region and the fact that the LDLa module at the N-terminus is key for receptor activation suggests that the hinge region in the RXFPs might not be involved in the binding and activation mechanism of these receptors.

The cognate ligands of RXFP1 and RXFP2 are H2 relaxin and INSL3, respectively. These peptides share structural similarity to insulin, where an A-chain and a B-chain are held together by two disulfide bonds (67, 68). Extensive studies of these two peptides conclude that the B-chain is essential for receptor binding [as reviewed in Ref. (10)]. The B-chain binding cassette of H2 relaxin is defined as RxxxRxxI/L, where x is any amino acid, and of INSL3 as HxxxRxxVR (68). Furthermore, a tryptophan residue located at the C-terminal of the B-chain is wrapped back around the structure of INSL3 and has been shown to be essential for binding and activation of its receptor, RXFP2 (69, 70). Binding to the LRR domains of RXFP1 and RXFP2 has been extensively studied by mutagenesis of both the receptors and H2 relaxin or INSL3. Previous modeling of the H2 relaxin–RXFP1 interaction has used RI as a template and subsequently, mutagenesis studies were performed to verify this model (71). The model of H2 relaxin–RXFP1 shows the conserved basic residues (Arg13 and Arg17) in the B-chain interact with acidic residues within the LRR6 and LRR8 of RXFP1, respectively. The other conserved hydrophobic residue (Ile20) within the B-chain is predicted to interact with a cluster of hydrophobic residues across LRR4-5. Hence, based on these data, it is proposed that H2 relaxin binds to the LRR domain at a 45^o^ angle across the face of LRR4–8. Scott et al. (72) also used the Nogo receptor as a template to model the INSL3–RXFP2 interaction, and given the expected structural similarity of RXFP1 and RXFP2 with the Nogo structure we present a model of RXFP1 (Figure 1C). The model of INSL3–RXFP2 concludes that the positively charged residues (Arg16 and Arg20) of INSL3 interact with negatively charged residues in LRR6 and 8 and the conserved hydrophobic residues in the B-chain of INSL3 (His12, Val19, and Trp27) with hydrophobic residues across LRR1–4. In this model, the B-chain of INSL3 requires a larger surface area than H2 relaxin and lies perpendicular to the LRRs.

The molecular details of how H2 relaxin and INSL3 bind and activate RXFP1 and RXFP2 are still ambiguous, despite extensive research. It is clear that a ligand-binding site is present in the LRR domain, but the relatively short LRRCT makes it unlikely to interact with the ligand in a type A LGR manner. While various receptor constructs show that neither the LDLa module nor the TM domain are required for high-affinity binding (29, 73), additional weak affinity binding sites for the ligand have been proposed for both RXFP1 and RXFP2 on the TM domain (73–75). Support for an interaction between the ligand and the TM domain includes experiments conducted on human relaxin 3 (H3 relaxin) (76), a homolog of H2 relaxin, and INSL3. H3 relaxin binds to both RXFP1 and RXFP3 (GPCR135). The latter lacks an ECD and so binding and activation is solely through the TM domain of RXFP3 (77). Taking advantage of the binding specificity of H3 relaxin for RXFP1 over RXFP2, ECD/TM domain chimeras of RXFP1/2 were constructed and tested for ligand binding and signaling (74). The chimera of the ECD of RXFP1 with the TM of RXFP2 binds H3 relaxin more weakly than to wild-type RXFP1, and signaling is reduced. On replacing the exoloop-2 of the TM domain in this construct with exoloop-2 of RXFP1, binding was similar to wild-type RXFP1 and signaling was fully restored, supporting an interaction by H3 relaxin with both the ECD and exoloop-2 of RXFP1. To further investigate the binding of H2 relaxin to the exoloops, exoloop-1 and exoloop-2 were engineered onto a soluble protein scaffold preserving the disulfide between exoloop-1 and -2 and therefore potentially creating a native-like structure of exoloop-2 (75). Using NMR spectroscopy and pull-down assays, specific interactions of H2 relaxin were observed to this scaffold, but not in a construct lacking the disulfide. These latter experiments show that the ligand binds to the exoloop-2, and also the importance of the integrity of the conformation of exoloop-2. Furthermore, mutation of a phenylalanine residue (equivalent to Phe564) to alanine within exoloop-2 showed loss of binding to H2 relaxin, and when this mutation was tested in the full-length receptor signaling was lost. Collectively, these data indicate that for type C LGRs, in contrast to type A and possibly type B, the ligands have binding sites on both the ECD and TM domain, where the latter are also essential for activation.

H2 relaxin binds to both RXFP1 and RXFP2, whereas INSL3 only binds RXFP2. To investigate if important differences lie within the LRR domain or involve the TM domain, a series of RXFP1/2 chimeras were prepared (78). These included constructs that swapped the TM domains or consisted of only the ECDs attached to single TM helices: referred to as 7BP or ECD-1 for the protein containing the ECD of RXFP1, and 8BP or ECD-2 for RXFP2 (8, 78). To test the contribution of the LRRs within the ECDs to ligand specificity and activation, residues within the LRRs of the RXFP1 constructs were swapped with LRRs of RXFP2 in order to gain INSL3 binding. Notably, in contrast to RXFP1, 7BP (ECD-1) binds INSL3, albeit more weakly than 8BP or RXFP2 but suggests that a binding site for INSL3 already exists in RXFP1. A high-affinity binding site for INSL3 was engineered into 7BP with as little as swapping a single LRR (specifically LRR1), and this binding was indistinguishable to that for 8BP or RXFP2. However, when the mutations that produce a high-affinity binding of INSL3 in 7BP were tested in full-length RXFP1, no gain in the binding of INSL3 was observed. As additional binding sites for INSL3 and H2 relaxin are proposed to be present on the TM domain (73–75), the TM domain of RXFP2 was also replaced on the RXFP1 construct that included the putative high-affinity binding site for INSL3. However, again binding or activation by INSL3 was not recovered. These data suggest that while clearly the LRR domain of these receptors harbors a ligand-binding site, additional binding features remain to be elucidated. Indeed, issues of the juxtaposition of the LRR with respect to the TM domain may sterically hinder INSL3 binding (78).

There is additional evidence of distinct differences in the mode of peptide binding to the RXFP1 and RXFP2 ECDs and the impact on receptor activation. Studies on synthetic H2 relaxin and INSL3 peptides with A-chain truncations or substitutions show distinct differences in the ability of the peptides to bind and activate RXFP1 and RXFP2. H2 relaxin peptides with truncations of the A-chain (79) or A-chain substitutions with other relaxin family peptide A chains (80) show loss of binding affinity in both RXFP1 and RXFP2 with parallel decreases in activation whereas truncations or alterations in the A-chain of INSL3 do not affect the high-affinity binding to RXFP2 (81–83) but abolish activation. These observations highlight that differences in the mode of ligand binding to these receptors exist and these modes have not been fully elucidated. Additionally, they highlight that H2 relaxin binds to RXFP2 in a manner different from the INSL3 mode and similar to the mode it binds to RXFP1.

### The LDLa module of type C LGRs

The presence of a unique N-terminal LDLa module distinguishes RXFP1 and RXFP2 from other LGRs, and indeed are the only GPCRs to contain this module (84). The LDLa module was first described as repeating units in the LDL receptor (85) and other related proteins (86) where they are involved in lipid metabolism. LDLa modules have since been described in a variety of proteins both as repeats and single domains in proteins with diverse functions, such as viral entry (87), breast cancer invasion and metastasis (88), and cell differentiation (89). LDLa modules are typically 4 kDa in size and have highly conserved structural features, including three disulfide bonds and an essential calcium ligation motif that contributes to maintaining overall fold and structure of the modules (85, 90, 91). The significance of the LDLa module in RXFP1 and RXFP2 was discovered during the characterization of splice variants of the receptors that lacked the LDLa modules (29). A naturally occurring splice variant of RXFP2 (LGR8-short) was identified and found to lack the LDLa module. While LGR8-short binds H2 relaxin and INSL3, no INSL3- or H2 relaxin-induced cAMP signaling was detected. This prompted the production of an engineered RXFP1 without the LDLa module (LGR7-short or RXFP1-short) and while it bound H2 relaxin equal to full-length RXFP1, no cAMP-induced signaling was detected (29). Recently, a panel of reporter genes was used to assess whether RXFP1 or RXFP2 without the LDLa module could signal through alternative GPCR signaling pathways other than those that signal through cAMP (92, 93). However, both RXFP1-short and RXFP2-short were unable to signal through any signaling pathway.

The structures of both the RXFP1 and RXFP2 LDLa modules have been solved using nuclear magnetic resonance (NMR) spectroscopy in an effort to understand the importance of specific residues (84, 93). While RXFP1 and RXFP2 have evolved to use the LDLa module for an essential role in signal activation, the molecular details are different between the two receptors. For example, chimeric RXPF2 (RXFP2–LB2), where the LDLa module is replaced with the second ligand-binding domain (LB2) of the low-density lipoprotein receptor (LDLr), showed some INSL3-induced cAMP activity (93), whereas a similar construct of RXFP1 (RXFP1–LB2) showed no significant H2 relaxin-induced cAMP activity (92). Adding back regions of the native RXFP2 LDLa sequence into RXFP2–LB2 did increase signaling of the module; however, this appeared to be due to reconstitution of the correct structure rather than specific side chain interactions. In comparison, in an attempt to rescue signaling in a RXFP1–LB2 chimera, the hydrophobic portions of the side chains of a cluster of residues (Leu7, Tyr9, and Lys17) were pinpointed to be essential, highlighting these residues may be involved in receptor activation (92). The capacity of these chimeric studies are insufficient to understand exactly the activation mechanism by the LDLa module, however, in a separate study using an engineered scaffold containing the extracellular loops of RXFP1, a weak interaction of the scaffold with the LDLa module was observed supporting the notion that it interacts with the TM domain for activation (75). Thus, in RXFP1 and RXFP2, the LDLa module is not involved in ligand binding, rather plays a crucial role in receptor activation by potentially interacting with the TM domain (29, 84, 92, 93). The LDLa module may therefore act as a tethered ligand that requires binding of H2 relaxin to mediate activation of the receptor; a mechanism which is distinct from the Type A LGRs two-step binding mechanism (47).

Joining the LDLa module to the LRR domain is a linker of variable length, 32 or 25 residues in human RXFP1 and RXFP2, respectively. This linker has been considered a simple tether with the function of intramolecular localization of the LDLa module to the TM domain for efficient activation. Swapping the LDLa module of RXFP2 onto RXFP1 resulted in loss of signaling, suggesting that the LDLa modules cannot be swapped; although in this study a large portion of the linker of RXFP2 was also swapped (94). However, in a second study, the LDLa modules of RXFP1 and RXFP2 were swapped, taking care not to alter the linker length or sequence, and these showed ligand-mediated activation (95). In this latter work, swapping the LDLa module of RXFP2 onto RXFP1, thus preserving the linker, LRR, and TM domains of RXFP1, showed wild-type H2 relaxin-induced cAMP activation. Importantly, maximum activation could not be obtained, suggesting that the LDLa module of RXFP2 could not make essential interactions with the TM domain of RXFP1 for full activation. These observations are consistent with site-directed mutagenesis experiments of the LDLa module in full-length RXFP1 (84, 92). When both the LDLa module and TM domain of RXFP2 were swapped onto RXFP1, maximum activation was achieved suggesting that the LDLa module was now acting as a full agonist and the interactions between the LDLa module and TM domain were fully restored. Swapping the LDLa module of RXFP1 onto RXFP2, thus preserving the linker, LRR, and TM domains of RXFP2, showed similar potency for both ligands (95). This may reflect the fact that H2 relaxin is a ligand of both RXFP1 and RXFP2 and the RXFP2–INSL3 evolved more recently (96). Thus the RXFP1 LDLa module may be equally efficacious on both RXFP1 and RXFP2. These results are in contrast to the LDLa-linker deletion where activation was lost (94) and challenge the notion that the linker is only a tether. Furthermore, these data suggest that the linker may play a role in activation akin to the hinge of the type A receptors. Importantly, a natural splice variant of RXFP1 where the LDLa module and the following linker residues are expressed as a soluble protein can antagonize the activity of H2 relaxin at RXFP1 supporting a functional role of the linker (29). Further research into whether the linker interacts with H2 relaxin or the TM domain is required.

## Conclusion

The LGR family has in common a LRR domain that serves as a ligand-binding site. From the point of view of mechanism, this is the only common feature of the three subtypes of receptors. At the extreme, the type B LGRs on binding ligand do not function through a canonical GPCR pathway by activation of either G-proteins or β-arrestin. When the ligand binds to type A LGRs, it undergoes a conformational change that enables an interaction between the ligand with the C-terminal hinge of the ECD, which is proposed to release the TM domain of the receptor from an inhibited state. Evidence presented for the type C LGRs, RXFP1 and RXFP2, shows that the true agonist of these receptors is the N-terminal LDLa module. Thus, it is hypothesized for these receptors that the binding of ligand results in a conformational change to the ECD to present the LDLa module to the TM domain for activation. Structures of the ECDs of type A and type B LGRs, free and in complex with ligand, suggest that conformational change of the LRR domain of the type C LGRs is unlikely. Given the size and structure of the ligands, H2 relaxin and INSL3, and the LDLa modules, it is difficult to envisage significant conformational changes to these molecules. Therefore, hypotheses of reorientation of the LDLa module or localization through modification of the structure of the linker that tethers the LDLa module to the LRR domain may be key to the activation process. Further research, including structure elucidation, is required to understand how the type C LGRs are activated.

## Conflict of Interest Statement

The authors declare that the research was conducted in the absence of any commercial or financial relationships that could be construed as a potential conflict of interest.
